# Editorial and Miscellaneous

**Published:** 1866-10

**Authors:** 


We purpose publishing, in successive numbers of the Journal, statistical statements of the deaths occurring in the Army of Tennessee, giving the names and commands to which they belonged, with summaries of the mean strength of the command, and per centage of mortality.

In this number we commence with General Bragg's command at Pensacola, and shall next take up the reports from the Army of Tennessee, under the same and subsequent commanders.

The Editors of this Journal are indebted to Dr. S. H. Stout, Director of Hospitals for that Army, for these reports and their arrangement for publication.

### LIST OF CONFEDERATE DEAD,

*Furnished by S. H. Stout, M. D., Professor of Surgical and Pathological Anatomy in the Atlanta Medical College, and late Medical Director of Hospitals of the Confederate Army of Tennessee.*

The following lists, as far as they go, are reliable and authentic. Many of the most valuable records of the office of Medical Director of Hospitals have been lost, captured or stolen. I have in possession many loose memoranda, and duplicates of reports, which, by much labor on my part, I hope to be able to collate and put in a valuable form for publication.

To rescue from oblivion the names of our dead braves, to gratify the friends of the deceased, and furnish the future historian of the war with such material as is in my hands, relating to the causes of death in the Confederate Armies, is suggested to my mind as a task, first demanding attention, in consequence of the form in which these records now exist.

These lists of dead will be furnished in the order of time as near as possible. The earliest reports now accessible to me, are those for the months of July, August, September, October, November and December, 1861, in the Confederate States Department of Alabama and West-Florida.

STATEMENT OF DEATHS in the Department of Alabama and West-Florida, commanded by General Braxton Bragg. From the Reports of Surgeon A. J. FOARD, Medical Director, Head-Quarters Pensacola, Florida.

*July*, 1861.

Thomas Shine, pvt., Fla. Regt.
E. M. Harrell, " " "
J. W. Cox, pvt., 9th Miss.
H. R. Hawkins, pvt., 7th Ala.
F. R. Moulton, pvt., La. Inft.
Morris Hurty, pvt., La. Inft.
John Hill, pvt., 1st Ala.
James Hardee, pvt., Fla. Rgt.
E. Mixon, " " "
M. Kelly, " " "
George Romberg, pvt., 10th Miss.
John Bossert, " " "
Joseph Graham, " 1st Ala.
J. L. Young, pvt., 9th Miss.
James Kerr, pvt., La. Inft.
—— Newsome, pvt., 1st Ala.
A. Joiner, pvt., 1st Ala.
W. J. Moore, pvt., Fla. Regt.
Joshua Wright, pvt., 1st Ala.
A. J. Cohen, pvt., 1st Ala.
John Seeley, pvt., Fla. Regt.
H. P. Morgan, pvt., 1st Ala.
J. B. Sheiffield, pvt., 7th Ala.
—— Collins, pvt., 10th Miss.
J. S. Bishop, pvt., 7th Ala.
John Milton, pvt., 1st Ala.
James Pew, pvt., 1st Ala.
Fabius Newlan, pvt., 10th Miss.
E. T. Benoit, sgt., " "
John S. Crowder, pvt., Fla. Regt.
H. Havard, " " "
Thomas Tate, corp. " "
W. E. Dennis, pvt., Ga. Battalion.

RECAPITULATION.

| | |
|---|---|
| Typhoid Fever, | 18 |
| Remittent Fever, | 4 |
| Intermittent Fever, | 1 |
| Phthisis Pulmonalis, | 1 |
| Dysenteria Acuta, | 2 |
| Dysenteria Chronica, | 1 |
| Rubeola, | 3 |
| Wounds of Abdomen, | 2 |
| Diarrhœa, | 1 |
| Total, | 33 |

SUMMARY.

Total cases received during the month, 3381; aggregate, 4125; sent to Gen'l Hospital, 411; returned to duty, 2246; furloughed, 53; discharged, 107; deaths, 33. Remaining on hand at the end of the month, sick, 1070; convalescent, 205. Total remaining, 1275. Strength of command: Officers, 331; enlisted men, 5823. Total strength, 6154.

Rates of deaths per 1000 of mean strength, 5.362.

---

*Deaths in August*, 1861, *at Pensacola and Vicinity.*

A. Robinson, pvt., 10th Miss.
R. M. Watson, " " "
Riley Earnest, " " "
W. H. Hall, pvt., Fla. Rgt.
R. H. Stephens, pvt., 9th Miss.
A. Coleman, pvt., La. Inft.
Patrick Murphy, pvt., La. Inft.
A. J. Williamson, pvt., 1st Ala.
S. P. S. Crockett, pvt., 9th Miss.
J. T. Harris, pvt., 7th Ala.
David Ballard, pvt., 7th Ala.
J. L. Stone, pvt., 1st Ala.
A. Warmick, pvt., 9th Miss.
G. E. Vaining, pvt., 1st Ala.
S. H. Raborne, pvt., 9th Miss.
James Coburn, pvt., 1st Ala.
W. G. Hines, pvt., Fla. Regt.
B. Warren, pvt., 1st Ala.
Stephen Underwood, pvt., La. Inft.
—— Scroggins, pvt., 7th Ala.

J. D. Rawls, pvt., 7th Ala.
William Porter, pvt., 1st Ala.
J. B. Phillips, pvt., 7th Ala.
—— Pettibone, pvt., La. Inft.
E. L. Clifton, pvt., 10th Miss.
Moses Norvoy, " " "
Green Heath, pvt., 7th Ala.
William Buchan, pvt., 1st Ala.
William Parris, pvt., 1st Ala.
J. B. Rhodes, pvt., 9th Miss.
Mich. Wheelan, pvt., La. Inft.
L. S. Rump, pvt., 1st Ala.
William Pithman, pvt., Fla Regt.
John Scott, pvt., La. Inft.
S. W. Alexander, pvt., 7th Ala.
—— Gammen, pvt., 1st Ala.
J. C. Newberry, " " "
Bryant Bates, " " "
T. J. Lawrence, " " "
Charles Sunis, " " "
Crittenden Coleman, pvt., 1st Fla.
R. H. Davis, " " "
M. Pitman, " " "
Daniel McKenna, " " "
R. J. Goodson, pvt., Prathville Dragoons.
William Wilson, pvt., 5th Ga.
William Dukes, pvt., " "
Michael Whalen, " " "
W. H. McMullen, corp., 10th Miss.

RECAPITULATION.

| | |
|---|---|
| Typhoid Fever, | 35 |
| Febris Continua Communis, | 2 |
| Pneumonia Typhoides, | 1 |
| Rubeola, | 2 |
| Dysenteria Acuta, | 6 |
| Dysenteria Chronica, | 1 |
| Suicidium, | 1 |
| Vulnus Punctum, | 1 |
| Total, | 49 |

SUMMARY.

Total cases received during the month, 3200; aggregate, 4475; sent to Gen'l Hospital, 340; returned to duty, 2558; furloughed, 313; discharged, 139; deserted, 7; died, 49. Remaining, sick, 575; convalescent, 394. Total remaining, 969. Mean strength of command: Officers, 369; enlisted men, 6271. Total mean strength, 6640.

Rates of deaths per 1000 of mean strength, 7.379.

---

*Deaths in September*, 1861, *at Pensacola and Vicinity.*

Albert Russell, pvt., 7th Ala.
Wiley Ludley, " " "
J. A. Caperton, " " "
J. Dowling, " " "
J. H. Hamilton, " " "
J. Ryals, pvt., Fla.
Thomas Cox, pvt., 9th Miss.
Otto Wonder, pvt., Ga. Battalion.
J. Clarke, pvt., La. Inft.
Richard Darby, pvt., 9th Miss.
J. D. Lipscomb, pvt., 7th Ala.
S. B. Herr, pvt., 9th Miss.
S. G. Egnor, pvt., 7th Ala.
T. M. Bishop, pvt., 9th Miss.
Henry Ming, pvt., 1st Ala.
D. Walker, pvt., 2nd L. Artillery.
W. M. Bounds, pvt., 7th Ala.
E. B. Whitley, pvt., 9th Miss.
J. H. T——, pvt., Ga. Batt.
E. J. Whitehurst, pvt., 7th Ala.
James Ford, pvt., 7th Ala.
W. J. Bryant, pvt., 7th Ala.
Thomas Maynard, pvt., 7th Ala.
H. C. Ingols, Lieut., 7th Ala.
W. H. Willis, pvt., 9th Miss.
W. J. Walea, pvt., Ga. Batt.
M. V. Wills, Chaplain, 9th Miss.
N. W. Braddy, pvt., 1st Ala.
Francis Smith, pvt., 5th Ga.
F. M. Meddlin, pvt., Fla. Regt.
S. O. Satterwhite, pvt., 5th Ga.
G. F. B. Thompson, pvt., 5th Ga.
John Sidney, pvt., C. S. Marines.

RECAPITULATION.

| | |
|---|---|
| Febris Typhoides | 21 |
| Febris Continua Communis | 2 |
| Dysenteria Acuta | 4 |
| Pneumonia | 2 |
| Pneumonia Typhoides | 1 |
| Rubeola | 1 |
| Gastritis | 1 |
| Vulnus Sclopeticum | 1 |
| Total | 33 |

SUMMARY.

Received during the month, 2590; aggregate treated, 3559; sent to Gen'l Hospital, 328; returned to duty, 2052; furloughed, 346; discharged, 46; deserted, 6; died, 33. Remaining, sick, 517; convalescent, 231. Total remaining, 748. Mean strength of the command: Officers, 368; enlisted men, 6453. Total mean strength, 6821.

Ratio of deaths per 1000 of mean strength, 4.867.

---

RECAPITULATION

*Of Deaths during the Quarter ending September* 30*th*, 1861, *at Pensacola and Vicinity.*

| | |
|---|---|
| Febris Typhoides | 74 |
| Febris Continua Communis | 4 |
| Febris Remittens | 4 |
| Febris Intermittnes | 1 |
| Phthisis Pulmonalis | 1 |
| Rubeola | 6 |
| Pneumonia | 2 |
| Pneumonia Typhoides | 2 |
| Dysenteria Acuta | 12 |
| Dysenteria Chronica | 2 |
| Diarrhœa | 1 |
| Wounds of Abdomen | 2 |
| Vulnus Punctum | 1 |
| Suicidium | 1 |
| Gastritis | 1 |
| Vulnus Sclopeticum | 1 |
| Total | 115 |

SUMMARY

*For the Quarter ending September* 30*th* 1861, *at Pensacola, Florida.*

| | |
|---|---|
| Remaining from previous month | 744 |
| Received during the Quarter | 9171 |
| Aggregate treated | 9915 |

